# The Analysis of Phenolic Compounds in Walnut Husk and Pellicle by UPLC-Q-Orbitrap HRMS and HPLC

**DOI:** 10.3390/molecules26103013

**Published:** 2021-05-19

**Authors:** Fang Sheng, Bangyan Hu, Qiang Jin, Jiangbo Wang, Cuiyun Wu, Zhengrong Luo

**Affiliations:** 1 Key Laboratory of Horticultural Plant Biology, Huazhong Agricultural University, Wuhan 430070, China; shengfang@webmail.hzau.edu.cn (F.S.); 18838933856@163.com (B.H.); 2 College of Plant Science, Tarim University, Alar 843300, China; jqzky@163.com (Q.J.); wangjiangbo@taru.edu.cn (J.W.); wcyby@163.com (C.W.)

**Keywords:** polyphenol, identification, quantification analysis, metabolic regulation

## Abstract

Husk and pellicle as the agri-food waste in the walnut-product industry are in soaring demand because of their rich polyphenol content. This study investigated the differential compounds related to walnut polyphenol between husk and pellicle during fruit development stage. By using ultra-high performance liquid chromatography-quadrupole-orbitrap (UHPLC-Q-Orbitrap), a total of 110 bioactive components, including hydrolysable tannins, flavonoids, phenolic acids and quinones, were tentatively identified, 33 of which were different between husk and pellicle. The trend of dynamic content of 16 polyphenols was clarified during walnut development stage by high-performance liquid chromatography (HPLC). This is the first time to comprehensive identification of phenolic compounds in walnut husk and pellicle, and our results indicated that the pellicle is a rich resource of polyphenols. The dynamic trend of some polyphenols was consistent with total phenols. The comprehensive characterization of walnut polyphenol and quantification of main phenolic compounds will be beneficial for understanding the potential application value of walnut and for exploiting its metabolism pathway.

## 1. Introduction

Walnut (*Juglans regia* L.) is a high nutritional nut due to the rich contents of unsaturated fatty acids, melatonin, vitamins, polyphenols and so on [1,2]. Among which, polyphenols are considered as to be important bioactive substance [2]. In addition, walnuts were also popular around the world for their health-promoting properties, such as reducing the risk of heart disease and cancer, improving blood circulation and reducing oxidative stress and inflammation [3,4,5]. Nowadays, walnut kernel is usually processed into various food; however, walnut husk and pellicle were usually directly discarded as waste or fuel, which caused resource waste and environmental pollution. Although walnut husk contained rich polyphenols [6], walnuts are mainly cultivated in order to obtain the kernels, and other parts of the nut, such as the shell and husk, are produced as waste during the fruit harvesting and processing [7,8]. Walnut pellicle only accounts for 5–8% of the whole walnut kernel [9] and is also the main source of walnut polyphenols, which could cause slight astringency and bitterness [10]; it is removed in the food processing, resulting in a waste of resources. At present, the research and development of agricultural waste with potential application value has been popularized and valued all over the world.

Walnut polyphenols contains phenolic acids, flavonoids, tannin and quinones [11]. Polyphenols exhibit a variety of activities, were the main compounds of walnut, which are important for human health [12]. However, there has been neither a systematic description of the chemical compound species nor a comprehensive comparison of the difference that exists in the polyphenol metabolites between the husk and pellicle of the walnut; the dynamic trend of the main compounds in the walnut polyphenol metabolism is especially unclear. Q-Orbitrap enables fast, sensitive and reliable detection and identification of small molecules without considering the relative ion abundance; it also has an extremely fast scanning speed, and the front body ions and product ions provide high-quality measurement results [11]. In addition, HPLC can be used for quick and accurate quantification the identification components, which has become one of the most economical technology [13]. Therefore, in order to explore the application value and main metabolites of the material, it is necessary to combine the new technology to comprehensively identify the polyphenol components in the husk and pellicle of the walnut.

In this study, Q-Orbitrap and HPLC were used for the systematic characterization and accurate quantification of the compounds in walnut husk and pellicle. Systematic characterization and quantification of polyphenol offers a comprehensive understanding of the potential application value of the walnut to improve recycling and help to explain its metabolism pathway.

## 2. Results and Discussion

### 2.1. Changes of Phenols and Flavonoid Content of Walnut Husk and Pellicle

**Figure 1:**
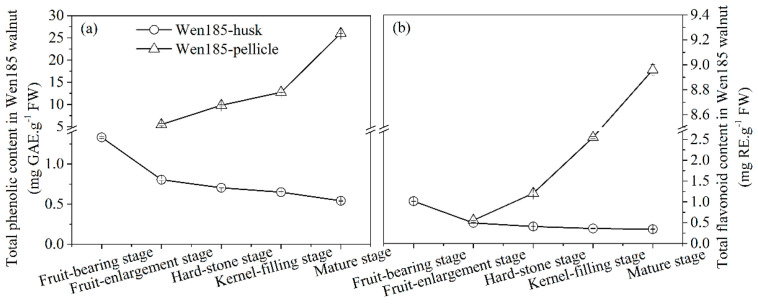
Changes of total phenol (**a**) and flavonoid (**b**) contents of walnut husk and pellicle.

The walnut husk and pellicle are a rich source of phenolic and flavonoid compounds [1]. In order to explore the change relationship between the total phenols (TPC) and total flavonoids (TFC) and the important polyphenol components, TPC and TFC were measured. TPC and TFC decreased continuously from fruit-bearing stage to mature stage in walnut husk but increased in pellicle (Figure 1). During the mature process, the range of TPC and TFC was 0.54–1.33 mg gallic acid equivalent/g fresh weight (GAE/g FW) and 0.34–1.01 mg rutin equivalent/g (RE/g) FW in walnut husk, respectively, which was similar to the results of Shi et al. [14]. For pellicle, the pellicle area increased gradually, but its weight gradually lightened with the growth cycle close to the mature stage; therefore, the accumulation of TPC and TFC showed an upward trend. The highest TPC and TFC was 26.02 mg GAE/g FW and 8.95 mg RE/g FW in walnut pellicle, respectively, compared with the phenolic contents in walnut reported in previous studies, such as leaf (0.34 mg GAE/g FW) [15], kernel (1.76 mg GAE/g FW) [16], shell (0.10 mg GAE/g FW) [16] and husk (1.14 mg GAE/g FW) [14], indicating that the pellicle was an abundant source of polyphenols.

### 2.2. Characterization of Phenolic Components in Walnut Husk and Pellicle

In this study, the UPLC system coupled with Q-Orbitrap system was used to analyze the phenolic compounds in husk and pellicle of the walnut, and a highly complex mixture of hydrolysable tannins, flavonoids and phenolic acids was contained. Moreover 110 compounds were successfully identified. Information on all compounds is listed in Table 1. Part of these compounds was reported in walnuts for the first time.

#### 2.2.1. Hydrolysable Tannins and Related Compounds

The ellagitannins (ET) contain the hexahydroxydiphenoyl (HHDP) group and monosaccharide [25], which were released on acid hydrolysis and spontaneously lactonized to ellagic acid (EA). The free EA was confirmed by [M−H]^−^ ion at *m/z* 300.99 (peak 67), and the fragment ions were confirmed at *m/z* 257.01 [M−H−CO_2_]^−^, *m/z* 229.02 [M−H−CO_2_−CO]^−^ and *m/z* 185.02 [M−H−2CO_2_−CO]^−^ [11]. In addition to EA, several monoglycosylated EA derivatives were observed in walnuts, such as EA pentoside (*m/z* 433.04, peak 58) (Supplementary Materials Figure S1) and EA hexaglucoside (*m/z* 463.05, peak 51). The ions at *m/z* 481.06, *m/z* 783.07 (peaks 1; 66), *m/z* 907.09 and *m/z* 951.08 (peaks 45; 53) were detected and identified as the hexahydroxydiphenolic acid (HHDP) glucose isomer, their intense fragment ion at *m/z* 301 [M−H−C_6_H_12_O_6_]^−^ indicated the loss of a glucose and the existence of an ellagic acid group, which has been reported as the main ET in walnuts [11]. The similar methyl ellagic acid derivatives can be identified by characteristic fragment ions at *m/z* 315.02 including methyl ellagic acid, for example methyl ellagic acid hexoside (*m/z* 477.06, peak 71), methyl ellagic acid pentose (*m/z* 447.06, peak 80).

A large number of gallotannins also presented in the extracts, ellagitannins were metabolically derived from gallotannins, mainly through C-C oxidative coupling of galloyl groups of pentagalloyl-glucose [23]. The characteristic of galloyl-glucose fragmentation was the generation of ion fragments by the continuous loss of gallic acid (170 Da) and galloyl (152 Da) [11]. The existence of [M−H]^−^ in 331.07, 483.08, 635.09 (Supplementary Materials Figure S2) and 787.10 indicated the presence of mono-, di-, tri- and tetra-galloyl glucose (peaks 3, 24, 57, 73, etc.), respectively. Several isomeric forms of these gallic acid esters of glucose with different retention times were found in our samples. Three or four galloyl acyl glucose also have found in walnut kernels and septum [11,17].

#### 2.2.2. Flavonoids

(+)-Catechin was identified as the main flavan-3-ol with [M−H]^−^ ion at *m/z* 289.07 and mass spectra/mass spectra (MS/MS) fragments at *m/z* 245.08, 205.05 and 123.04, which were consistent with the results reported by Gómez-Caravaca et al. [26]. Moreover, (−)-epicatechin (peak 41) shared the same molecular ion with catechin. The [M−H]^−^ ion (peak 56) at *m/z* 441.08 with the fragment ions of *m/z* 289.07, which was generated due to the breaking of the ester bond, was tentatively identified as (−)-epicatechin gallate isomer (Supplementary Materials Figure S3). It was also found in walnut kernel [17]. Procyanidins were polymers of catechin and epicatechin, with the oligomeric procyanidin monomers linking mainly through C4→C8 or C4→C6 bonds [19]. In this study, four kinds of oligomers of procyanidins were detected, with degree of polymerization (DP) from 2 to 4, and all belonged to type B. The [M−H]^−^ ion of procyanidin dimer (18) and trimer (27) were at *m/z* 577.14 and 865.20 [11], respectively. The [M−2H]^2−^ ion of procyanidin tetramer (peak 29) at *m/z* 576.13 was observed (Supplementary Materials Figure S4). It has been reported that procyanidins with DP from 4 to 6 in walnut kernel [23], however, more species of oligomeric procyanidins were found in this study.

Flavonol has been concerned for its free radical scavenging and anti-inflammatory activities and its ability to resist to cardiovascular disease [27,28]. The peak 65 was detected at *m/z* 615.09, with its fragments at *m/z* 463.13 (M−H−152, loss of galloyl group) and *m/z* 301.00 (M−H−162, loss of hexosyl group). It was tentatively identified as quercetin galloyl-hexoside [11]. Peak 79 at *m/z* 433.08, including the 301.00 fragment (M−H−132, loss of pentose unit), was identified as quercetin pentoside (Supplementary Materials Figure S5). Peak 91 at *m/z* 463.09 produced a fragment ion at *m/z* 301.03 (M−H−162, loss of hexoside moiety), and 300.02 (H rearrangement) was identified as quercetin-*O*-glucoside [20]. Myricetin-*O*-hexoside (*m/z* 479.08, peak 60) showed fragment ions at *m/z* 317.03 and 316.02, corresponding to the loss of a hexoside moiety (−162 Da). Peak 94 was identified as kaempferol-rhamnoside, with fragments at *m/z* 285.04 and [M−H]^−^ ion at *m/z* 431.10, and it was also detected in the walnut septum [11].

Flavanol, also known as dihydroflavonol, is a subclass of polyphenols that is negatively related to diabetes in animal and in vitro models. Taxifolin (*m/z* 303.05, peak 68) showed fragment ions at *m/z* 285.04, 177.02 and 125.02; the ion at *m/z* 285 was due to the loss of a water molecule (−18 Da), whereas, at *m/z* 177 and 125, the ions correspond to cleavage of the C ring [18] (Supplementary Materials Figure S6). The astilbin (*m/z* 449.11, peak 75) was preliminarily identified; the MS^2^ spectrum of *m/z* 449.11 produced ions at *m/z* 303.05, 285.04 and 151.00, and those at *m/z* 303 and 285 were generated by the loss of a rhamnose (−146 Da) and consecutive loss of a water molecule (−18 Da) [18], which has been identified in grapes and was identified in walnuts for the first time.

Eriodictyol and its glycoside derivatives are the main flavanones in the walnut husk and pellicle. Eriodictyol (*m/z* 287.06, peak 98) showed products at *m/z* 151.00 and 135.04 formed by an retro diels-alder (RDA) type fragmentation in the C ring. The MS^2^ spectrum of eriodictyol-*O*-hexoside (*m/z* 449.11, peak 88) produced ions at *m/z* 287.06 generated by the loss of a hexosyl moiety (−162 Da); this is the first report of eriodictyol-*O*-hexoside in walnuts.

#### 2.2.3. Phenolic Acid and Derivatives

Phenolic acid and its derivatives are easy to cleavage CO_2_ from carboxylic acid [11]. The neutral loss of CO_2_ was observed in MS/MS data of gallic acid (*m/z* 169.01, peak 4), protocatechuic acid (*m/z* 153.02, peak13), *p*-hydroxybenzoic acid (*m/z* 137.02, peak 20), vanillic acid (*m/z* 167.03, peak 25), caffeic acid (*m/z* 179.03, peak 30), syringic acid (*m/z* 197.04, peak 32) and *p*-coumaric acid (*m/z* 163.03, peak 70). The peak 10 was detected at *m/z* 353.09 with its fragments at *m/z* 191.06 and 179.03, which can be identified as caffeoylquinic acids (neochlorogenic acid). The [M−H]^−^ ion at *m/z* 337.09 (peak 26) generated fragments ion at *m/z* 163.04, *m/z* 173.05, 119.05 and 93.03, which was corresponding to the loss of quinic acid, according to published literature, it was identified as coumaroylquinic acid [11]. Based on the [M−H]^−^ ion and the MS^2^ spectra showed the characteristic fragmentation involving in cleavage of the hexosyl moiety (−162 Da) [29], hexoside derivatives of gallic acid were detected in walnut extract, for example gallic acid hexoside (*m/z* 331.07, peak 6) showed product ion at *m/z* 271 and 211, probably generated the hexose moiety fragmentation (−60 Da) and removal of two formaldehyde (CH_2_O) groups in the glucose moiety, respectively.

#### 2.2.4. Identification of Quinones

The fragmentation of quinones was produced by cleaving the substituents or eliminating the carbon on the benzene ring; Quinones were identified by the characteristic ions produced by the neutral loss of H_2_O, CO or CH_2_ due to the cleavage of hydroxy group on the benzene ring [11]. For example, compound 72 was tentatively identified as naphthalenediol isomers on the molecular ions at *m/z* 161.06 and fragment ion at *m/z* 133.07, 115.06, 105.07 and 91.06 (Supplementary Materials Figure S7).

### 2.3. Comparative Analysis of Components in Walnut Husk and Pellicle

Among the characterized active components, 86 and 101 species were identified in the walnut husk and pellicle (Supplementary Materials Figure S8), respectively, with 33 different components in total (Table 2). Further analysis showed that the most diverse type was hydrolysable tannin (17 kinds), followed by flavonoids (5 kinds). Compared with the pellicle, naphthoquinones and flavonoids mainly appeared in husk, which was consistent with the main components detected in walnut husk in previous studies [30]. However, most of the flavanols and condensed tannins appeared in the pellicle; flavanol is a precursor for the synthesis of condensed tannins, which was consistent with the astringency in pellicle [31].

### 2.4. Content Variation of Main Phenolic Compounds during Walnut Ripening

Sixteen polyphenols were chosen for further quantification. In order to determine the polyphenols quickly and accurately, the composition and pH of the mobile phase, flow rate, injection volume, column temperature and elution condition were optimized. The calibration curves and results of individual components of walnut polyphenols are shown in Supplementary Materials Table S1 and Figure S9.

Although Q-Orbitrap is highly sensitive and suitable for identification compounds, due to its relatively high instrument cost, HPLC was used to quantify the dynamic changes of several main phenolics. During walnut growth and development stage, in the husk, the content of neochlorogenic acid, catechin, chlorogenic acid, myricetin and juglone showed a downward trend; *p*-hydroxybenzoic acid, *p*-coumaric acid and vanillic acid showed an upward trend; rutin, syringic acid, ferulic acid, *o*-coumaric acid, caffeic acid and quercetin showed a W-type curve, all of their content was highest in fruit-bearing stage; and caffeic acid was only detected in the mature stage. In the pellicle, most of the polyphenol components showed an increasing trend; however, the content of juglone was higher in the fruit enlargement stage; it then decreased and rose again in the hard-stone stage and slightly decreased in the mature stage. The change trend of ferulic acid was contrary to juglone; quercetin content increased from fruit enlargement stage to kernel filling stage and then decreased; neochlorogenic acid and vanillic acid were only detected in the hard-stone stage and mature stage (Table 3).

The content of polyphenols was significantly different between husk and pellicle, and it was higher in pellicle. At present, there are few studies on the trend of walnut components. In Shi’s study [14], a series of time nodes were chosen, and no trend was found. In this study, only the key nodes were studied, and we found a certain regularity trend between main components and the TPC/TFC (Table 4). In husk, the changes of gallic acid, neochlorogenic acid, catechin, rutin, myricetin and juglone were highly correlated with the changes of TPC/TFC, while in pellicle, the changes of *p*-coumaric acid, rutin and myricetin were highly correlated with the changes of TPC/TFC. In addition, the growth and development dynamics of walnuts in different environments are not identical, which also affect the content and composition of walnut polyphenols. Although the results in this paper are not exactly same as those of Shi et al., the main conclusion is identical, at the fruit bearing and early development stage, TPC and most polyphenols were the highest in husk, young fruit can be used as the first choice to study phenolic component.

There are relatively few studies on walnut components, and no reports about its tannin biosynthesis pathway, which limits the improvement of walnut genetic breeding. Therefore, to screen the key metabolism of walnut tannins, explore their relationship with the synthesis of walnut astringent substances and their regulatory of tannin metabolism for a deeper understanding of the regulatory mechanism of walnut tannin synthesis, and clarify its biology synthetic pathways, it is of great significance to enrich walnut genetics and breeding.

## 3. Materials and Methods

### 3.1. Materials

“Wen 185” were collected from the Germplasm Resources Base in Tarim University (44°55′ N, 81°28′ E), Xinjiang Uygur Autonomous Region, China. Five stages’ fruits were collected from April to September, namely fruit-bearing, fruit-enlargement, hard-stone, kernel-filling and mature stages; the green husk and pellicle of the seed were separated and treated with liquid nitrogen, and then they were stored in a freezer (−80 °C) until analysis.

### 3.2. Standards and Reagents

All standards were purchased from Shanghai Yuanye Biotechnology Co., Ltd. (Shanghai, China). HPLC-grade acetonitrile, methanol, formic acid and acetic acid were purchased from Fisher Scientific (Fairlawn, NJ, USA). Deionized water was prepared by using a Milli-Q system (Merck Millipore, Burlington, MA, USA).

### 3.3. Polyphenols Extraction

TPCs and TFCs were measured according to the method of Slinkard and Singleton [32] and Jia et al. [33], with some modifications. The extraction method is as follows: 2 g of each fresh mixed sample was grounded into powder and extracted with 40 mL of 50% methanol under ultrasonic conditions at 50 °C temperature for 45 min. The solution was centrifuged at 13,000 rpm for 10 min, and we collected the supernatants. Then, using a vacuum rotary evaporator, the residue was frozen in a lyophilizer (Labconco, Kansas, MO, USA). All samples were dissolved with 10 mL pure methanol, filtered (0.22 μm) and stored in a refrigerator (−20 °C) [14].

### 3.4. Preparation Standard Solution

Sixteen reference standards were accurately weighed and dissolved in the mixed standard working solutions at concentrations of 5.0–10.0 mg/mL for quantitative analysis. For the construction of calibration curves, 16 standard stock solutions were mixed and further diluted with 50% methanol to produce a series of standard solutions at the concentration range of 0.25–625.0 μg/mL. All solutions were stored at 4 °C, in a refrigerator, before analysis.

### 3.5. Qualitative and Quantitative Components in Walnut Husk and Pellicle

The identification samples were prepared by the following method of Liu et al. [11]. The extraction solution in 3.3 was diluted to 50% methanol for the quantification analysis.

A Thermo U-3000 HPLC system (Thermo Scientific, Waltham, MA, USA) and Q Exactive Orbitrap MS system (Thermo Scientific, Waltham, MA, USA) were used to identify the components in walnut. A Hypersil GOLD C18 column (100 × 2.1 mm, 1.8 μm) was applied for chromatographic separation, the condition of chromatography and mass spectrometry refer to the method used in Liu et al. [11]. The mass spectrometer was operated in both positive- and negative-ion modes. MS detection conditions were set as follows: spray voltage, +3.5 kV/−3.2 kV; capillary temperature, 320 °C; sheath gas, 35 arb; AUX gas, 10 arb; AUX gas heater temperature, 350 °C; s-lens RF level, 50; scan range, *m/z* 100−1500; resolution, 70,000 (MS^1^) and 17,500 (MS^2^ ); stepped normalized collision energy (NCE), 20, 40 and 60%; injection time, 3 s; frequency of Orbitrap mass calibration, once a week. The compounds library was build; a high-throughput search and a manual search were performed by matching with the library and fragment-ion information.

A Waters high-performance liquid chromatography system (Waters, Milford, MA) and an Agilent SB-C18 column (250 × 4.6 mm, 5 μm) were used to detect walnut polyphenols. The column temperature was 30 °C, and the injection volume was 10 μL. The flow rate was 1.0 mL/min; the mobile phase consisted of water containing 0.5% acetic acid as eluent A and methanol as eluent B. The program of 280 nm detection wavelength was as follows: 0–10 min, 10–17.5% B; 10–11 min, 17.5–20% B; 11–35 min, 20–37% B; 35–38 min, 37–50% B; 38–40 min, 50–10% B (for gallic acid, neochlorogenic acid, (+)-catechin, *p*-hydroxybenzoic acid, chlorogenic acid, vanillic acid, caffeic acid, epicatechin, syringic acid, *p*-coumaric acid, ferulic acid and *o*-coumaric acid). The gradient elution program of 251 nm wavelength was as follows: 0–5 min, 10–50% B; 5–15 min, 50–60% B; 15–25 min, 60–70% B; 25–30 min, 70–10% B (for rutin, myricetin, quercetin and juglone).

### 3.6. Statistical Analysis

All analyses were performed in triplicate. Quantification data were expressed as means ± standard deviation (SD). One-way analysis of variance (ANOVA) was carried out on the quantitative data, using Microsoft Excel 2010 at a significance level of *p* < 0.05. MS data were processed by using Compound Discoverer 3.0 (Thermo Fisher, San Jose, CA, USA).

## 4. Conclusions

In this experiment, the components and contents of polyphenols in walnut husk and pellicle were analyzed by Q-Orbitrap combined with HPLC. This was the first time that the compositions of the walnut husk and pellicle were characterized and compared. A total of 110 components were successful characterized, and 33 different components were found in the husk and pellicle; most was hydrolysable tannin. The obtained compound information can be used as a reference for rapid analysis and identification compounds, which will not only support its efficient development and reuse but also benefit to explore hidden application value. In addition, 16 polyphenols were quantified; the dynamic trend analysis of the main phenolic content which was contained in walnut husk and pellicle provides a basis for exploring the differences between their metabolic pathways and analyzing their regulatory networks.

## Figures and Tables

**Table 1:** Characterization of the components from walnut extracts by UHPLC–Q-Orbitrap HRMS.

No.	RT (min)	Formula	Ion Mode	Measured Mass (*m/z*)	MS/MS Fragments (*m/z*)	Error (ppm)	Compound Identification	Reference	Classification	Walnut	
										Husk	Pellicle
1	0.744	C_20_H_18_O_14_	M−H	481.06317	301.000, 275.020, 229.014, 257.010	1.666	HHDP-glucose isomer	[11]	Hydrolysable Tannins	−	+
2	0.895	C_7_H_12_O_6_	M−H	191.05539	85.028, 93.033, 87.008, 59.013, 173.045	−3.772	quinic acid	[17]	Hydrolysable Tannins	+	+
3	1.162	C_13_H_16_O_10_	M−H	331.06760	169.014, 271.046, 211.025, 125.023	1.620	monogalloyl-glucose isomer	[11]	Hydrolysable Tannins	+	+
4	1.307	C_7_H_6_O_5_	M−H	169.01352	125.023, 107.013	−4.264	gallic acid	[11]	Phenolic Acid	+	+
5	1.361	C_8_H_8_O_4_	M−H	167.03427	123.044, 139.003	−4.243	hydroxymandelic acid	[18]	Phenolic Acid	+	+
6	2.073	C_13_H_16_O_10_	M−H	331.06760	169.014, 125.023, 271.046, 211.025	1.620	gallic acid hexoside	[19]	Phenolic Acid	−	+
7	2.699	C_13_H_16_O_9_	M−H	315.07281	108.021, 152.011	2.110	protocatechuic acid-*O*-hexoside	[20]	Phenolic Acid	+	+
8	3.749	C_8_H_8_O_4_	M+H	169.04951	111.044, 65.039, 125.060, 169.050, 151.039, 93.034	−3.284	isovanillic acid		Phenolic Acid	+	+
9	3.786	C_7_H_6_O_3_	M−H	137.02347	108.021, 93.033	−2.861	protocatechualdehyde	[18]	Phenolic Acid	+	−
10	3.971	C_16_H_18_O_9_	M−H	353.08832	191.056, 135.044, 179.034	1.467	neochlorogenic acid	[11]	Phenolic Acid	+	+
11	4.090	C_9_H_6_O_3_	M+H	163.03888	163.039, 107.049, 95.050	−3.377	7-hydroxycoumarine		Other	+	+
12	4.102	C_14_H_18_O_10_	M−H	345.08307	168.006, 124.016	1.040	methyl galloyl hexoside	[18]	Hydrolysable Tannins	+	+
13	4.171	C_7_H_6_O_4_	M−H	153.01846	109.028, 81.033	−5.665	protocatehuic acid	[18]	Phenolic Acid	+	+
14	4.270	C_15_H_20_O_10_	M−H	359.09897	197.045, 153.055	1.667	dimethyl galloyl hexoside	[18]	Hydrolysable Tannins	+	+
15	4.447	C_15_H_18_O_9_	M−H	341.08826	177.055, 135.044	1.662	caffeic acid hexoside II	[21]	Phenolic Acid	+	+
16	4.448	C_8_H_8_O_5_	M−H	183.02930	168.006, 124.016	−3.240	methyl gallate	[19]	Other	+	+
17	4.470	C_13_H_15_O_8_	M−H	299.07767	137.024	1.872	hydroxybenzoyl hexoside	[20]	Phenolic Acid	+	+
18	4.485	C_30_H_26_O_12_	M−H	577.13678	125.024, 289.072, 407.078, 425.089, 109.029, 137.024	2.841	B-type procyanidin dimer isomer	[11]	Flavonoid	−	+
19	4.814	C_21_H_22_O_12_	M−H	465.10535	303.051, 285.039, 201.113	3.239	epicatechin-3-*O*-glucoside isomer	[18]	Flavonoid	+	+
20	4.911	C_7_H_6_O_3_	M−H	137.02348	93.033, 108.021, 137.024	−6.763	*p*-hydroxybenzoic acid	[19]	Phenolic Acid	+	+
21	4.912	C_15_H_14_O_6_	M−H	289.07224	109.029, 245.082, 123.044, 125.024, 203.071, 137.024, 151.039, 205.050, 97.028	1.644	(+)-catechin	[11]	Flavonoid	+	+
22	4.930	C_14_H_10_O_9_	M−H	321.02539	169.014, 125.024	0.594	*m*-digallate	[18]	Phenolic Acid	+	+
23	4.930	C_10_H_6_O_3_	M−H	173.02376	173.024, 145.029, 117.034	3.225	2-hydroxy-1, 4-naphthoquinone		Quinones	+	+
24	5.099	C_20_H_20_O_14_	M−H	483.07886	169.014, 125.024, 271.047, 211.025, 313.057, 331.068	1.733	digalloyl-glucose isomer	[11]	Hydrolysable Tannins	−	+
25	5.102	C_8_H_8_O_4_	M−H	167.03427	152.011, 108.021, 123.044	−4.243	vanillic acid	[17]	Phenolic Acid	+	+
26	5.175	C_16_H_18_O_8_	M−H	337.09344	163.039, 119.049, 173.045, 93.033	1.646	3-p-coumaroylquinic acid	[11]	Phenolic Acid	+	+
27	5.199	C_45_H_38_O_18_	M−H	865.19952	125.024, 289.072, 407.078, 151.039	1.149	B-type procyanidin trimer isomer	[11]	Flavonoid	−	+
28	5.266	C_10_H_10_O_3_	M+H	179.07021	133.065, 147.044	−0.415	juglanoside isomer	[18]	Quinones	+	+
29	5.352	C_60_H_50_O_24_	M−2H	576.12830	289.072, 287.057, 407.079	2.171	B-type procyanidin tetramer isomer	[11]	Flavonoid	+	+
30	5.452	C_9_H_8_O_4_	M−H	179.03435	135.044, 107.049	−3.533	caffeic acid	[17]	Phenolic Acid	+	+
31	5.473	C_27_H_22_O_18_	M−H	633.07495	301.000, 275.020, 125.023, 229.014, 257.010	2.561	galloyl-HHDP-glucose	[11]	Hydrolysable Tannins	−	+
32	5.701	C_9_H_10_O_5_	M−H	197.04512	182.022, 123.008, 166.998, 153.055, 138.031	−2.130	syringic acid	[17]	Phenolic Acid	+	+
33	5.777	C_41_H_28_O_26_	M−H	935.08185	275.020, 301.000, 229.014, 257.010, 299.020	2.500	galloyl-bis-HHDP-glucose	[18]	Hydrolysable Tannins	+	−
34	5.887	C_15_H_22_O_5_	M−H	281.13980	237.150, 171.118, 123.080, 189.129	1.284	dihydrophaseic acid	[18]	Other	+	+
35	5.927	C_10_H_10_O_3_	M−H	177.05504	177.055, 159.044, 149.060, 131.049	−3.793	isosclerone isomer	[18]	Other	+	+
36	5.951	C_10_H_8_O_3_	M+H	177.05447	177.055, 149.060, 131.049, 121.065, 159.044	−3.977	5-hydroxy-1, 4-naphthaoquinon	[22]	Quinones	+	+
37	5.961	C_16_H_18_O_9_	M−H	353.08832	135.044, 179.034, 191.056	1.467	chlorogenic acid	[18]	Phenolic Acid	+	+
38	6.177	C_15_H_18_O_8_	M−H	325.09341	265.072, 119.049, 163.039, 205.050, 235.061, 145.029	1.613	coumaric acid hexoside isomer	[11]	Phenolic Acid	+	+
39	6.185	C_10_H_10_O_2_	M+H	163.07530	131.049, 103.055, 163.075	−3.421	methyl cinnamate		Phenolic Acid	+	+
40	6.205	C_11_H_12_O_5_	M+H	225.07591	175.039, 91.055, 119.049, 147.044, 207.101	−2.477	sinapinic acid		Phenolic Acid	+	+
41	6.342	C_15_H_14_O_6_	M−H	289.07224	109.029, 245.082, 123.044, 125.024, 203.071, 137.024, 151.039	1.644	(−)-epicatechin	[18]	Flavonoid	+	+
42	6.344	C_8_H_8_O_3_	M−H	151.03922	136.016, 108.021, 123.045	−5.594	vanillin	[18]	Other	+	+
43	6.387	C_21_H_22_O_13_	M−H	481.09924	169.014, 125.024, 300.999	1.004	galloyl methylgalloyl dexoyhexoside isomer	[18]	Hydrolysable Tannins	−	+
44	6.400	C_16_H_18_O_8_	M+H	339.10748	177.055, 145.028, 321.097, 117.034	0.097	trihydroxynaphthaline glucoside	[18]	Quinones	+	+
45	6.510	C_40_H_28_O_25_	M−H	907.08575	301.000, 275.020, 783.070	1.769	heterophylliin E isomer	[23]	Hydrolysable Tannins	−	+
46	6.546	C_10_H_6_O_3_	M+H	175.07520	119.086, 147.080, 129.070	−2.280	juglone	[22]	Quinones	+	+
47	6.571	C_14_H_18_O_9_	M−H	329.08835	167.034, 123.044	3.307	vanillic acid hexoside		Phenolic Acid	+	+
48	6.743	C_21_H_10_O_13_	M−H	469.00540	425.016, 301.000	1.155	valoneic acid dilactone	[23]	Other	−	+
49	6.854	C_10_H_8_O_3_	M−H	175.03929	147.044, 131.049	−4.391	7-hydroxy-methylcoumarin	[18]	Phenolic Acid	+	−
50	6.920	C_21_H_22_O_12_	M−H	465.10403	285.041	0.418	quercetin-3-*O*-galactopyranoside isomer	[18]	Flavonoid	+	+
51	6.953	C_20_H1_6_O_13_	M−H	463.05298	300.999, 301.072	3.729	ellagic acid hexoside isomer	[11]	Phenolic Acid	−	+
52	7.085	C_9_H_10_O_4_	M−H	181.05002	166.027, 151.003, 123.008	−3.379	syringaldehyde	[18]	Phenolic Acid	+	+
53	7.103	C_41_H_28_O_27_	M−H	951.07581	301.000, 275.020, 783.071, 299.992, 229.014, 257.010, 907.087	1.362	trigalloyl-HHDP-glucose	[11]	Hydrolysable Tannins	−	+
54	7.135	C_9_H_8_O_3_	M−H	163.03932	119.049, 93.033	−4.527	*o*-coumaric acid	[18]	Phenolic Acid	+	+
55	7.299	C_27_H_22_O_19_	M−H	649.06903	300.999	1.203	valoneoyl-glucose	[19]	Hydrolysable Tannins	+	+
56	7.384	C_22_H_18_O_10_	M−H	441.08365	289.072, 109.029, 245.082, 125.024, 203.071, 151.039, 205.050	2.128	(−)-epicatechin 3-*O*-gallate	[11]	Flavonoid	−	+
57	7.460	C_27_H_24_O_18_	M−H	635.09070	169.014, 125.024, 483.079, 313.057, 465.068, 211.025	2.705	trigalloyl-glucose isomer	[11]	Hydrolysable Tannins	−	+
58	7.527	C_19_H_14_O_12_	M−H	433.04199	299.992, 301.000, 229.014	1.737	ellagic acid pentoside isomer	[11]	Phenolic Acid	−	+
59	7.547	C_34_H_26_O_22_	M−H	785.08685	301.000, 275.020, 229.014, 125.023, 257.010, 169.014	3.265	digalloyl-HHDP-glucose	[11]	Hydrolysable Tannins	−	+
60	7.696	C_21_H_20_O_13_	M−H	479.08334	316.023, 317.031	0.487	myricetin-*O*-hexoside	[20]	Flavonoid	+	+
61	7.802	C_21_H_20_O_13_	M+H	481.09705	319.045, 153.018, 85.029, 91.039	−1.152	myricetin-3-*O*-beta-*D*-galactopyranoside	[22]	Flavonoid	+	+
62	7.835	C_20_H_20_O_11_	M−H	435.09390	151.003, 285.041, 107.013	1.431	taxifolin-3-*O*-arabinofuranoside isomer	[18]	Flavonoid	+	−
63	7.874	C_9_H_10_O_3_	M+H	167.07022	167.070, 123.044, 149.096, 121.029	−3.286	ethyl paraben		Phenolic Acid	+	+
64	7.878	C_9_H_10_O_3_	M−H	165.05498	121.029, 108.021	−4.438	hydroxyphenyl-propionic acid	[18]	Phenolic Acid	+	+
65	8.121	C_28_H_24_O_16_	M−H	615.10004	300.028, 301.036, 271.025, 169.014, 125.024	1.441	quercetin galloyl hexoside isomer	[11]	Flavonoid	+	+
66	8.140	C_34_H_24_O_22_	M−H	783.07117	301.000, 275.020, 229.014, 257.009	3.227	pedunculagin/casuariin isomer (bis-HHDP-glucose)	[11]	Hydrolysable Tannins	−	+
67	8.298	C_14_H_6_O_8_	M−H	300.99945	229.015, 283.997, 257.010, 185.024	1.531	ellagic acid	[11]	Phenolic Acid	+	+
68	8.320	C_15_H_12_O_7_	M−H	303.05136	125.024, 285.041, 177.019	1.980	taxifolin isomer	[18]	Flavonoid	+	−
69	8.356	C_10_H_10_O_4_	M−H	193.05016	175.039, 147.044, 178.027, 131.049	−2.457	ferulic acid	[18]	Phenolic Acid	+	+
70	8.429	C_9_H_8_O_3_	M−H	163.03925	119.049, 93.033	−4.995	*p*-coumaric acid	[17]	Phenolic Acid	+	+
71	8.502	C_21_H_18_O_13_	M−H	477.06006	299.992, 315.016	1.150	methyl ellagic acid hexoside	[19]	Phenolic Acid	+	+
72	8.704	C_10_H_8_O_2_	M+H	161.05965	133.065, 105.070, 115.054, 91.055	−0.394	naphthalened iol isomer	[18]	Quinones	+	+
73	8.718	C_34_H_28_O_22_	M−H	787.10297	169.014, 125.024, 617.080, 465.069, 313.057	3.845	tetragalloyl-glucose isomer	[11]	Hydrolysable Tannins	−	+
74	8.779	C_21_H_20_O_12_	M−H	463.08939	271.025, 151.003, 178.998	3.764	myricitrin	[18]	Flavonoid	+	+
75	9.040	C_21_H_22_O_11_	M−H	449.10910	151.003, 285.041, 125.023, 107.013, 303.051, 178.998	0.379	astilbin isomer	[18]	Flavonoid	+	+
76	9.116	C_15_H_10_O_6_	M+H	287.05499	287.055, 213.055, 153.018, 121.029, 241.050	−1.973	kaempferol	[22]	Flavonoid	+	+
77	9.153	C_15_H_10_O_6_	M−H	285.04068	175.039, 133.029, 151.003	0.772	luteolin	[18]	Flavonoid	+	+
78	9.235	C_41_H_30_O_26_	M−H	937.09729	301.000, 257.010, 635.090, 785.086	2.754	tellimagrandin II	[11]	Hydrolysable Tannins	−	+
79	9.630	C_20_H_18_O_11_	M−H	433.07828	300.028, 301.036, 271.025, 151.003	2.788	quercetin pentoside isomer	[18]	Flavonoid	+	−
80	9.679	C_20_H_16_O_12_	M−H	447.05780	315.015	1.231	methyl ellagic acid pentose	[19]	Phenolic Acid	−	+
81	9.742	C_23_H_22_O_12_	M−H	489.10464	175.039, 169.014, 271.047, 125.023, 313.057, 229.051	1.645	3’-*O*-acetylquercitrin isomer	[18]	Flavonoid	+	+
82	9.844	C_23_H_22_O_12_	M+H	491.11835	153.018, 201.054, 297.060	−0.128	trihydroxynaphthalene-*O*-(*O*-trihydroxybenzoyl) glucoside	[18]	Quinones	+	+
83	9.910	C_21_H_20_O_11_	M−H	447.09402	300.028, 301.036, 271.025, 255.030, 151.003, 243.030, 178.998	1.666	quercitrin	[18]	Flavonoid	+	+
84	9.960	C_21_H_22_O_10_	M−H	433.11514	271.062, 151.003, 119.049, 177.019	3.846	naringenin-7-*O*-glucoside isomer	[18]	Flavonoid	+	+
85	10.131	C_48_H_32_O_31_	M−2H	551.03961	301.000, 169.014, 125.023, 275.020	1.437	calamanin A isomer	[18]	Hydrolysable Tannins	−	+
86	10.167	C_9_H_16_O_4_	M−H	187.09717	125.096, 97.065, 168.888	−2.202	azelaic acid	[18]	Other	+	−
87	10.239	C_22_H_22_O_12_	M−H	477.10464	169.014, 125.024	1.687	isorhamnetin-3-*O*-glucoside isomer	[18]	Flavonoid	+	−
88	10.423	C_21_H_22_O_11_	M−H	449.10995	287.057	2.282	eriodictyol-*O*-hexoside	[20]	Flavonoid	+	+
89	10.526	C_26_H_18_O_16_	M−H	585.05341	301.000, 433.040	2.061	ellagic acid galloyl pentose	[19]	Hydrolysable Tannins	−	+
90	10.676	C_28_H_24_O_14_	M−H	583.11023	300.028, 271.025, 255.030, 301.035, 151.003	1.560	quercetin-*O*-(p-hydroxy)benzoyl-hexoside	[18]	Flavonoid	+	+
91	10.904	C_21_H_20_O_12_	M−H	463.08951	301.036, 151.003, 178.998	2.846	quercetin-*O*-glucoside	[20]	Flavonoid	+	+
92	10.909	C_25_H_26_O_12_	M−H	517.13623	175.039	2.109	dihydroxynaphthol-*O*-[*O*-(dimethoxy-hydroxybenzoyl)] glucopyranoside	[18]	Quinones	+	+
93	11.014	C_23_H_20_O_12_	M+H	489.10269	327.050, 265.049, 237.055, 309.039	−0.140	jugnaphthalenoside A	[18]	Quinones	+	+
94	11.015	C_20_H_16_O_11_	M−H	431.09912	285.041, 255.030, 284.033, 227.035	1.742	kaempferol-rhamnoside	[11]	Flavonoid	+	+
95	11.085	C_37_H_30_O_16_	M−H	729.14972	125.023, 169.014, 407.078, 289.072	4.964	(epi)catechin-(4,8’)-3’-*O*-galloyl-(epi)catechin	[18]	Flavonoid	−	+
96	11.231	C_16_H_20_O_9_	M−H	355.10428	175.040, 134.036, 160.016	2.333	juglanoside D isomer	[18]	Quinones	+	+
97	11.312	C_28_H_35_NO_13_	M−H	592.20520	241.108, 403.162, 343.140	3.741	glansreginin A	[23]	Other	−	+
98	11.812	C_15_H_12_O_6_	M−H	287.05640	135.044, 151.003	0.998	eriodictyol	[20]	Flavonoid	+	+
99	11.813	C_21_H_24_O_10_	M−H	435.13080	167.034, 125.023, 123.044, 273.078, 119.049	2.593	phlorizin	[18]	Other	+	+
100	12.186	C_15_H_20_O_10_	M−H	359.09894	197.045	1.582	syringic acid hexoside	[24]	Phenolic Acid	+	+
101	12.229	C_15_H_10_O_7_	M−H	301.03577	151.003, 107.013, 178.998	1.317	quercetin	[23]	Flavonoid	+	+
102	13.474	C_15_H_12_O_5_	M−H	271.06180	93.033, 177.019, 119.049, 107.013, 151.003	2.243	naringenin	[18]	Flavonoid	+	+
103	14.008	C_15_H_14_O_5_	M−H	273.07748	151.003, 189.055, 125.024, 167.034, 123.044, 119.049	2.359	phloretin	[18]	Other	+	+
104	14.024	C_9_H_10_O_2_	M−H	149.05992	149.009, 105.070	3.698	hydrocinnamic acid		Other	+	+
105	14.582	C_10_H_12_O	M+H	149.09618	149.096, 105.070, 79.055, 65.039	−3.715	cuminaldehyde		Other	−	+
106	14.630	C_16_H_12_O_6_	M−H	299.05661	256.039, 227.035, 284.033	1.672	kaempferide isomer	[18]	Flavonoid	+	+
107	17.493	C_20_H_20_O_7_	M+H	373.12814	343.081, 183.029, 271.059, 297.075	−1.492	tangeritin		Flavonoid	+	+
108	20.835	C_13_H_11_O_9_	M−H	311.25992	149.096	2.436	caftaric acid	[20]	Phenolic Acid	+	+
109	21.734	C_27_H_30_O_16_	M−H	609.51074	255.233, 271.047	1.936	rutin	[17]	Flavonoid	+	+
110	23.493	C_10_H_6_O_4_	M−H	189.01877	161.024, 117.034	−2.972	dihydroxy-naphthoquinone isomer	[18]	Quinones	+	−

Note: A plus sign (+) and a minus sign (−) represent the presence or absence of the data in walnut husk or pellicle at the corresponding level, respectively.

**Table 2:** Classification of different components in husk and pellicle of walnut.

Classification	Husk	Pellicle
Hydrolyzable tannins		digalloyl-glucose, ellagic acid galloyl pentose, galloyl methylgalloyl dexoyhexoside isomer, tetragalloyl-glucose, trigalloyl-glucose, calamanin A isomer, digalloyl-HHDP-glucose, ellagic acid hexoside isomer, ellagic acid pentoside isomer, galloyl-bis-HHDP-glucose, galloyl-HHDP-glucose, heterophylliin E isomer, methyl ellagic acid pentose, Tellimagrandin II, trigalloyl-HHDP-glucose, HHDP-glucose isomer, pedunculagin/casuariin isomer (bis-HHDP-glucose)
Flavonoids	quercetin pentoside isomer, taxifolin, taxifolin-3-*O*-arabinofuranoside isomer	isorhamnetin-3-*O*-glucoside isomer, (−)-epicatechin 3-*O*-gallate
Phenolic acid	protocatechualdehyde, 7-hydroxy-methylcoumarin	gallic acid hexoside
Quinones	dihydroxy-naphthoquinone isomer	
Terpenoids		cuminaldehyde, valoneic acid dilactone
Condensed tannins		B-type procyanidin dimer isomer, procyanidin trimer, (epi)catechin-(4, 8’)-3’-*O*-galloyl-(epi)catechin

**Table 3:** The contents of 16 polyphenols in walnut husk and pellicle during development by HPLC.

	Husk					Pellicle			
Compounds	FBS	FES	HSS	KFS	MS	FES	HSS	KFS	MS
Gallic acid	16.52 ± 0.49 ^a^	2.88 ± 0.30 ^c^	2.72 ± 0.08 ^c^	4.24 ± 0.08 ^b c^	1.11 ± 0.01 ^d^	77.08 ± 6.11 ^d^	214.28 ± 6.62 ^c^	353.38 ± 12.26 ^a^	325.78 ± 1.93 ^b^
Neochlorogenic acid	89.09 ± 3.90 ^a^	34.61 ± 1.25 ^b^	23.42 ± 1.50 ^d^	17.34 ± 0.62 ^c^	13.65 ± 0.58 ^e^	<LOD ^a^	<LOD ^a^	374.60 ± 20.48 ^b^	530.92 ± 18.87 ^a^
Catechin	162.37 ± 3.30 ^a^	93.97 ± 0.99 ^b^	68.37 ± 0.29 ^c^	63.30 ± 2.53 ^c^	54.71 ± 4.50 ^c^	587.29 ± 47.49 ^b^	787.23 ± 44.09 ^a b^	942.33 ± 53.93 ^a^	302.39 ± 6.83 ^c^
*p*-Hydroxybenzoic acid	10.44 ± 0.87 ^a^	0.30 ± 0.01 ^c^	0.26 ± 0.01 ^d^	1.01 ± 0.03 ^b c^	1.10 ± 0.11 ^b^	152.98 ± 3.36 ^b^	174.55 ± 7.91 ^a^	171.54 ± 18.13 ^a^	195.56 ± 8.64 ^a^
Chlorogenic acid	2.41 ± 0.14 ^a^	1.61 ± 0.08 ^b^	1.18 ± 0.01 ^c^	<LOD ^a^	<LOD ^a^	15.96 ± 1.32 ^c^	30.25 ± 0.01 ^b^	41.36 ± 1.29 ^a^	<LOD ^a^
Vanillic acid	30.25 ± 0.12 ^a^	0.25 ± 0.04 ^e^	0.57 ± 0.02 ^d^	0.94 ± 0.08 ^c^	1.47 ± 0.04 ^b^	<LOD ^a^	77.09 ± 6.11 ^a^	47.24 ± 0.60 ^b^	<LOD ^a^
Caffeic acid	<LOD ^a^	<LOD ^a^	<LOD ^a^	<LOD ^a^	0.66 ± 0.01 ^a^	13.42 ± 0.86 ^c^	24.83 ± 0.68 ^b^	60.12 ± 2.51 ^a^	80.15 ± 2.75 ^a^
Epicatechin	<LOD ^a^	<LOD ^a^	<LOD ^a^	<LOD ^a^	1.31 ± 0.08 ^a^	<LOD ^a^	<LOD ^a^	<LOD ^a^	<LOD ^a^
Syringic acid	8.23 ± 0.05 ^a^	2.18 ± 0.15 ^c^	5.46 ± 0.05 ^b^	1.61 ± 0.09 ^c^	2.07 ± 0.22 ^c^	113.94 ± 5.77 ^c^	154.54 ± 2.94 ^b^	210.84 ± 9.07 ^a^	214.35 ± 8.67 ^a^
*p*-Coumaric acid	0.76 ± 0.06 ^a^	<LOD ^a^	0.12 ± 0.00 ^d^	0.16 ± 0.00 ^c^	0.29 ± 0.00 ^b^	2.99 ± 0.26 ^d^	5.42 ± 0.32 ^c^	7.42 ± 0.17 ^b^	12.72 ± 0.02 ^a^
Ferulic acid	2.14 ± 0.10 ^a^	0.37 ± 0.01 ^c^	0.46 ± 0.04 ^b c^	0.30 ± 0.03 ^d^	0.65 ± 0.03 ^b^	1.49 ± 0.03 ^c^	4.70 ± 0.06 ^a^	1.85 ± 0.12 ^c^	4.24 ± 0.11 ^b^
*o*-Coumaric acid	0.37 ± 0.01 ^a^	0.17 ± 0.01 ^d^	0.29 ± 0.01 ^c^	0.19 ± 0.00 ^d^	0.33 ± 0.01 ^b^	0.52 ± 0.03 ^c^	1.50 ± 0.05 ^b c^	1.52 ± 0.10 ^b^	1.77 ± 0.06 ^a^
Rutin	54.39 ± 2.53 ^a^	6.21 ± 0.75 ^b c^	8.33 ± 0.95 ^b^	1.52 ± 0.36 ^d^	2.63 ± 0.04 ^c^	308.37 ± 10.35 ^d^	1023.80 ± 7.62 ^c^	1626.42 ± 7.61 ^b^	3623.17 ± 32.49 ^a^
Myricetin	326.56 ± 5.48 ^a^	64.16 ± 7.63 ^b^	61.21 ± 12.95 ^b^	34.20 ± 5.14 ^c^	34.98 ± 2.37 ^c^	3366.58 ± 9.27 ^c^	3086.85 ± 37.77 ^d^	4375.62 ± 83.86 ^b^	8453.82 ± 383.06 ^a^
Quercetin	23.32 ± 0.79 ^a^	6.02 ± 0.04 ^c^	9.99 ± 0.44 ^b^	5.97 ± 0.44 ^c^	6.48 ± 0.20 ^c^	24.52 ± 2.14 ^b^	34.84 ± 0.75 ^a^	18.04 ± 1.97 ^c^	4.54 ± 0.07 ^d^
Juglone	15.42 ± 0.46 ^a^	5.44 ± 0.20 ^b^	1.88 ± 0.13 ^c^	0.79 ± 0.01 ^d^	0.57 ± 0.01 ^e^	2.13 ± 0.17 ^a^	1.37 ± 0.04 ^b^	2.10 ± 0.08 ^a^	1.78 ± 0.01 ^a^

Note: FBS, fruit-bearing stage; FES, fruit enlargement stage; HSS, hard-stone stage; KFS, kernel filling stage; MS, mature stage; LOD, limit of detection. The number of independent original samples is 3; data are shown as mean ± standard deviation (μg/g fresh weight). Different letters for the same phenolic compound indicate significant differences among the different developmental stages (*p* < 0.05).

**Table 4:** Pearson-correlation study between individual phenolic and TPC/TFC in husk and pellicle of walnut.

	Husk		Pellicle	
Compounds	TPC	TFC	TPC	TFC
Gallic acid	0.962 **	0.969 **	0.716	0.62
Neochlorogenic acid	0.996 **	0.999 **	0.889	0.871
Catechin	0.994 **	0.991 **	−0.632	−0.721
*p*-Hydroxybenzoic acid	0.925 *	0.959	0.946	0.895
Chlorogenic acid	0.884 *	0.849	−0.571	−0.675
Vanillic acid	0.940 *	0.971 **	−0.319	−0.455
Caffeic acid	−0.48	−0.36	0.921	0.886
Epicatechin	−0.48	−0.36	-	-
Syringic acid	0.83	0.842	0.805	0.729
*p*−Coumaric acid	0.783	0.847	0.994 **	0.969 *
Ferulic acid	0.903 *	0.949	0.512	0.448
*o*−Coumaric acid	0.439	0.532	0.766	0.661
Rutin	0.968 **	0.987 **	0.999 **	0.983 *
Myricetin	0.975 **	0.992 **	0.968 *	0.993 **
Quercetin	0.933 *	0.953 *	−0.84	−0.884
Juglone	0.990 **	0.993 **	−0.164	−0.085
TFC	0.991 **		0.989 *	

* There was significant correlation at 0.05 level (bilateral). ** There was significant correlation at 0.01 level (bilateral). Symbol “-” means no date.

## Data Availability

The data presented in this study are available on request from the corresponding author.

## Supplementary Materials

The following are available online, Figure S1: Extracted ion chromatograms (EIC) and tandem mass spectra (MS^2^) of ellagitannins identified in the extract of walnut. * The peak numbers are according to Table 1, Figure S2: Extracted ion chromatograms (EIC) and tandem mass spectra (MS^2^) of gallotannins identified in the extract of walnut, Figure S3: Extracted ion chromatograms (EIC) and tandem mass spectra (MS^2^) of flavonoids identified in the extract of walnut, Figure S4: Extracted tandem mass spectra (MS^2^) of peak 29 identified in the extract of walnut, Figure S5: Extracted ion chromatograms (EIC) and tandem mass spectra (MS^2^) of flavonol identified in the extract of walnut, Figure S6: Extracted ion chromatograms (EIC) and tandem mass spectra (MS^2^) of flavanol identified in the extract of walnut, Figure S7: Extracted ion chromatograms (EIC) and tandem mass spectra (MS^2^) of quinones identified in the extract of walnut, Figure S8: Total-ion chromatograms in negative mode of walnut husk and pellicle extracts, Figure S9: HPLC chromatogram of the standard optimized system, Table S1: Calibration curves, Retention time, Linear ranges, LODs and LOQs for 16 analytes.

## Sample Availability

Samples of the compounds are available from the authors.
