# Supplementary material for: The Analysis of Phenolic Compounds in Walnut Husk and Pellicle by UPLC-Q-Orbitrap HRMS and HPLC
Source: Molecules. 2021 May 19;26(10):3013. doi: 10.3390/molecules26103013 (PMC8158686; doi:10.3390/molecules26103013)
Supplement: Supplementary file 1 [file molecules-26-03013-s001.zip › molecules-1173493-SI.pdf]

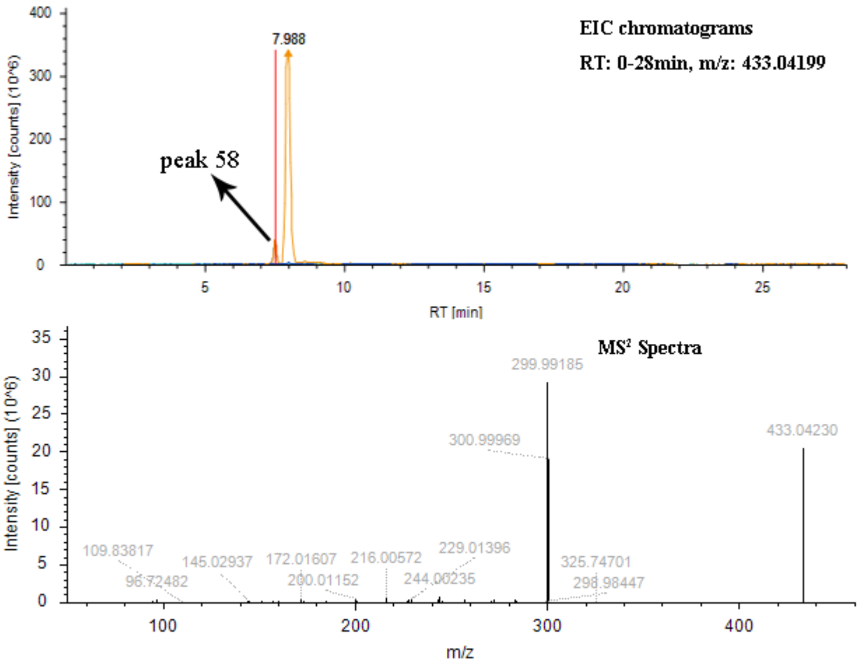

Figure S1. Extracted ion chromatograms (EIC) and tandem mass spectra (MS^2) of ellagitannins identified in the extract of walnut. * The peak numbers are according to Table 1.

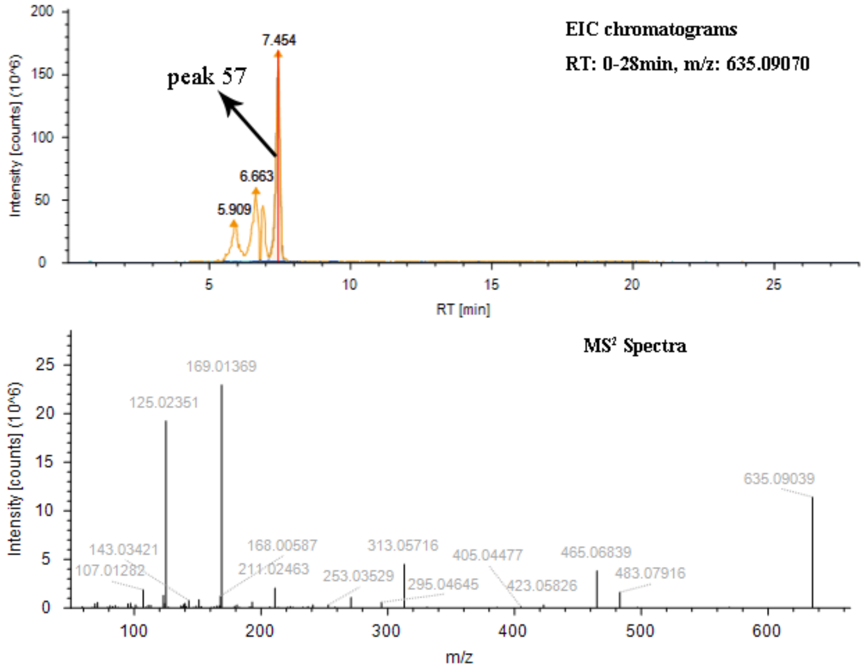

Figure S2. Extracted ion chromatograms (EIC) and tandem mass spectra (MS^2) of gallotannins identified in the extract of walnut.

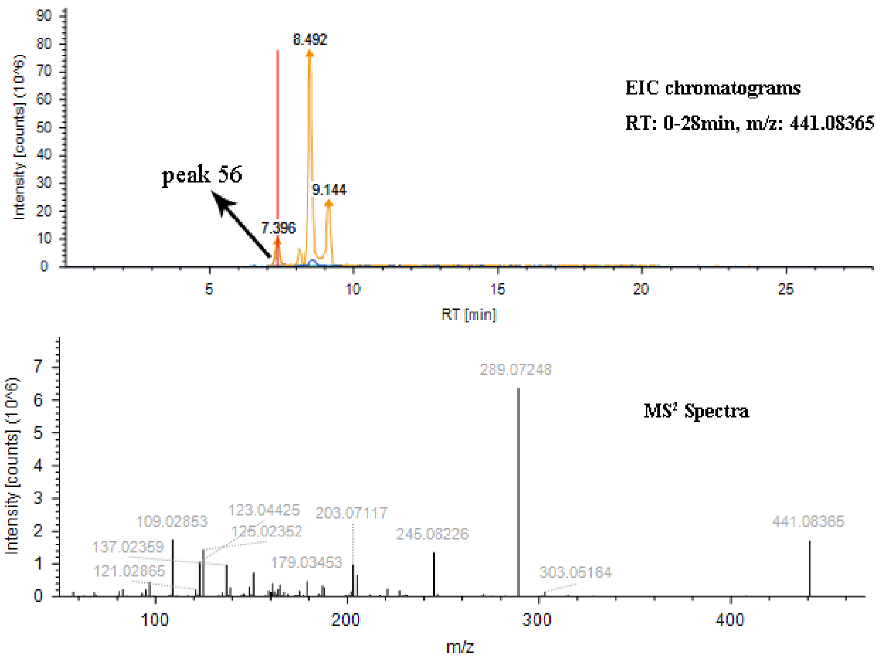

Figure S3. Extracted ion chromatograms (EIC) and tandem mass spectra (MS^2) of flavonoids identified in the extract of walnut.

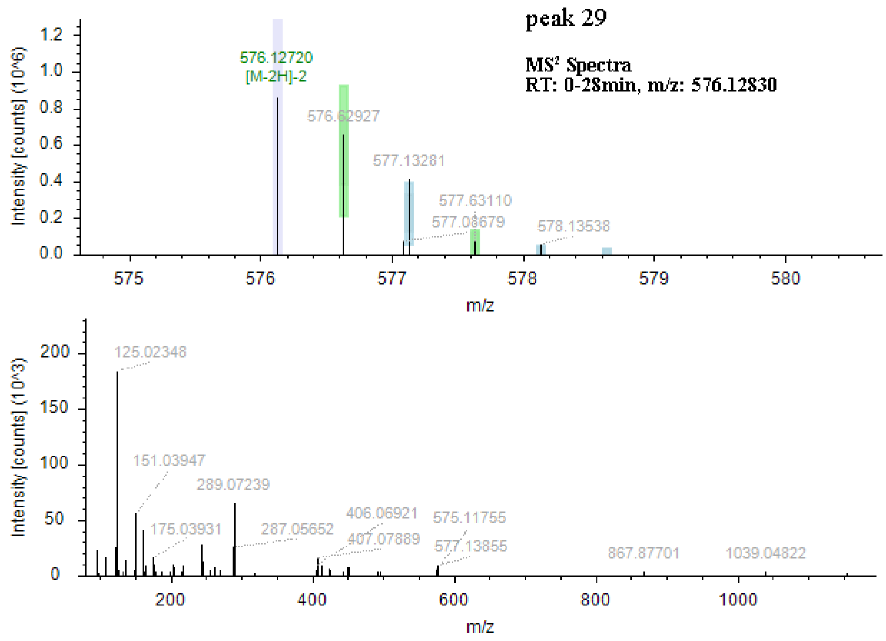

Figure S4. Extracted tandem mass spectra (MS^2) of peak 29 identified in the extract of walnut.

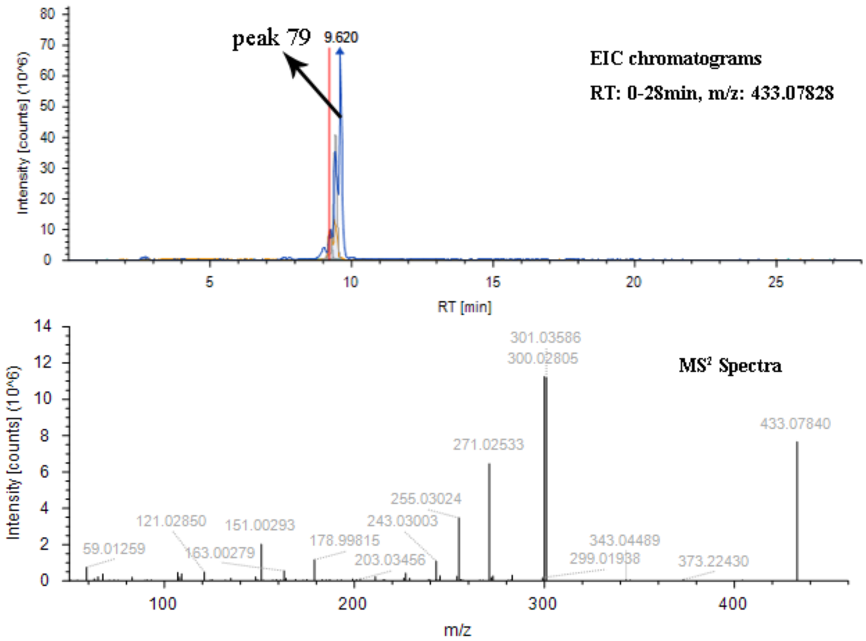

Figure S5. Extracted ion chromatograms (EIC) and tandem mass spectra (MS^2) of flavonol identified in the extract of walnut.

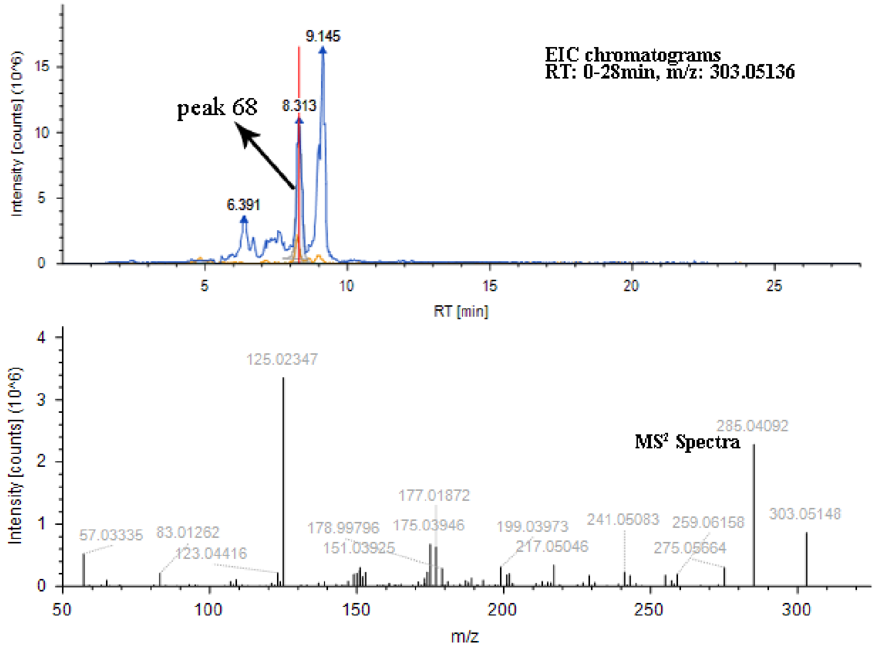

Figure S6. Extracted ion chromatograms (EIC) and tandem mass spectra (MS^2) of flavanol identified in the extract of walnut.

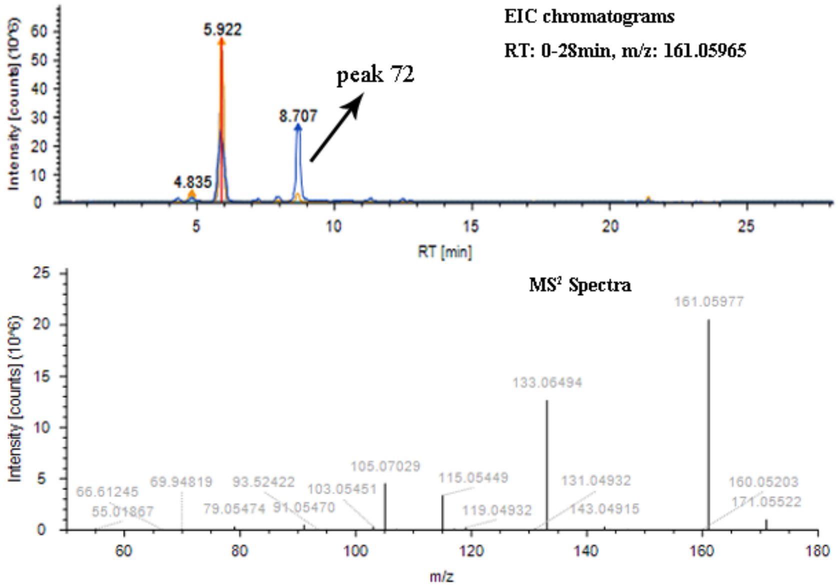

Figure S7. Extracted ion chromatograms (EIC) and tandem mass spectra (MS^2) of quinones identified in the extract of walnut.

Table S1. Calibration curves, Retention time, Linear ranges, LODs and LOQs for 16 analytes

Compound	Calibration curve	R^2	Retention time	UV max (lambda in nm)	Linear ranges (µg/mL)	LOQ (µg/ml)	LOD (µg/ml)
gallic acid	y = 0.0004x - 0.0037	R^2= 0.9992	4.537	280	10.00-625.00	1.20	0.36
neochlorogenic acid	y = 0.0007x - 0.0038	R^2= 0.9993	9.592	280	10.00-625.00	0.52	0.16
chlorogenic acid	y = 0.0016x - 0.0029	R^2= 0.9996	14.470	280	10.00-500.00	0.86	0.26
catechin	y = 0.0008x - 0.0045	R^2= 0.9988	15.428	280	5.00-625.00	0.54	0.16
p-hydroxybenzoic acid	y = 0.0007x - 0.0047	R^2= 0.9986	16.959	280	10.00-625.00	0.83	0.25
vanillic acid	y = 0.0007x - 0.0042	R^2= 0.999	19.426	280	10.00-625.00	1.00	0.30
caffeic acid	y = 0.0003x - 0.0006	R^2= 0.9997	19.904	280	5.00-500.00	0.40	0.12
epicatechin	y = 0.0016x + 0.0014	R^2= 0.9988	22.004	280	10.00-500.00	0.83	0.25
syringic acid	y = 0.0004x - 0.0010	R^2= 0.9998	22.454	280	5.00-500.00	0.38	0.11
p-coumaric acid	y = 0.0002x - 0.0005	R^2= 0.9998	28.144	280	5.00-500.00	0.23	0.07
ferulic acid	y = 0.0004x - 0.0011	R^2= 0.9997	31.285	280	5.00-500.00	0.43	0.13
o-coumaric acid	y = 0.0002x - 0.0030	R^2= 0.9994	35.168	280	10.00-625.00	0.31	0.09
rutin	y = 0.0013x - 0.0336	R^2= 0.9971	10.410	251	50.00-5000.00	1.88	0.56
myricetin	y = 0.0123x - 0.1200	R^2= 0.9947	11.741	251	50.00-5000.00	11.21	3.36
quercetin	y = 0.0062x + 0.0025	R^2= 0.9981	14.710	251	50.00-500.00	11.23	3.37
juglone	y = 0.0007x + 0.0004	R^2= 0.9985	16.448	251	10.00-500.00	1.10	0.33

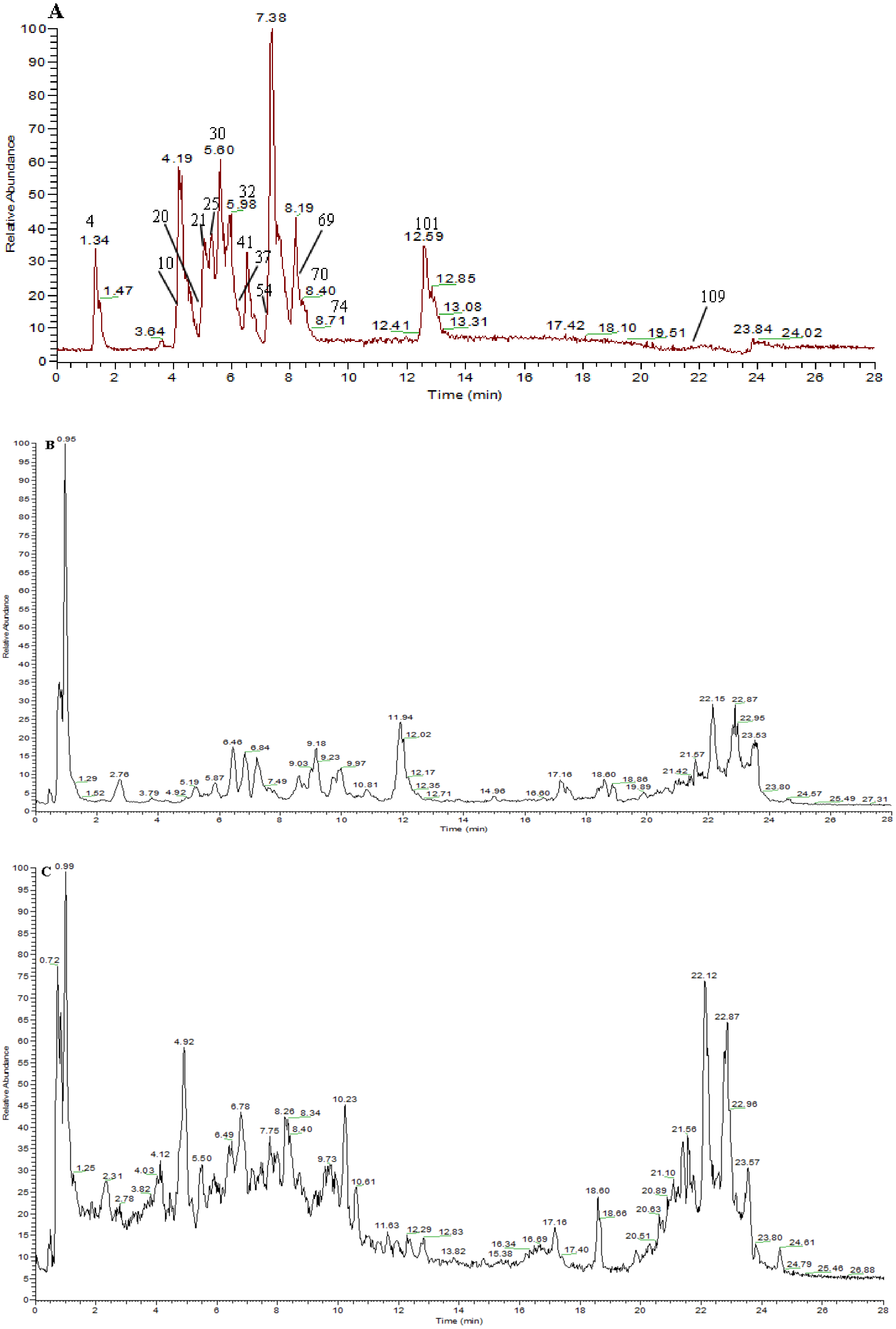
A
Relative Abundance
Time (min)
7.38
4.19
30
5.60
32
5.98
25
21
20
10
41
37
54
4
1.34
1.47
3.64
8.19
69
70
8.40
74
8.71
101
12.59
12.85
13.08
13.31
12.41
17.42
18.10
19.51
109
23.84
24.02
B
0.95
6.46
6.84
9.18
9.03
9.23
9.97
7.49
10.81
11.94
12.02
12.17
12.35
12.71
1.29
1.52
2.76
3.79
4.92
5.19
5.87
14.96
16.60
17.16
18.60
18.86
19.89
21.42
21.57
22.15
22.87
22.95
23.53
23.80
24.57
25.49
27.31
C
0.99
0.72
4.92
1.25
2.31
2.78
3.82
4.03
4.12
5.50
6.49
6.78
7.75
8.26
8.34
8.40
9.73
10.23
10.61
11.63
12.29
12.83
13.82
15.38
16.34
16.69
17.16
17.40
18.60
18.66
20.51
20.63
20.89
21.10
21.56
22.12
22.87
22.96
23.57
23.80
24.61
24.79
25.46
26.88

Figure S8. Total-ion chromatograms in negative mode of walnut husk and pellicle extracts. (A: Chromatogram of quantitative standards; B: Chromatogram of walnut husk; C: Chromatogram of walnut pellicle)

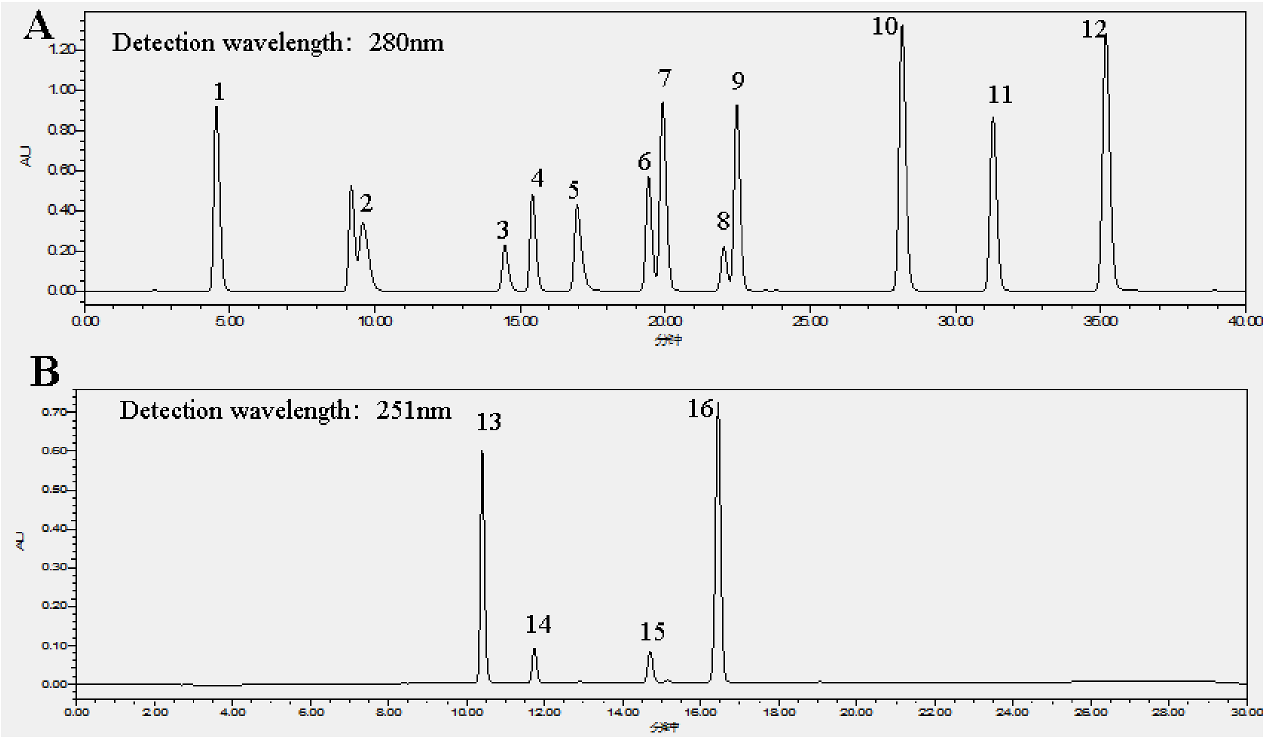

Figure S9. HPLC chromatogram of the standard optimized system (Note: 1. gallic acid; 2. neochlorogenic acid; 3. catechin; 4. *p*-hydroxybenzoic acid; 5. chlorogenic acid ; 6. vanillic acid; 7. caffeic acid; 8. epicatechin; 9. syringic acid; 10. *p*-coumaric acid; 11. ferulic acid; 12. *o*-coumaric acid; 13. rutin; 14. myricetin; 15. quercetin; 16. juglone)
